# Bone Status in Obese, Non-diabetic, Antipsychotic-Treated Patients, and Effects of the Glucagon-Like Peptide-1 Receptor Agonist Exenatide on Bone Turnover Markers and Bone Mineral Density

**DOI:** 10.3389/fpsyt.2018.00781

**Published:** 2019-01-28

**Authors:** Robert Eriksson, Brian V. Broberg, Pelle L. Ishøy, Nikolaj Bak, Ulrik B. Andersen, Niklas R. Jørgensen, Filip K. Knop, Bjørn H. Ebdrup

**Affiliations:** ^1^Mental Health Centre Glostrup, Copenhagen University Hospital, Glostrup, Denmark; ^2^Department of Disease Systems Biology, Faculty of Health and Medical Sciences, Novo Nordisk Foundation Center for Protein Research, University of Copenhagen, Copenhagen, Denmark; ^3^Center for Neuropsychiatric Schizophrenia Research, Center for Clinical Intervention and Neuropsychiatric Schizophrenia Research, Mental Health Centre Glostrup, Copenhagen University Hospital, Glostrup, Denmark; ^4^Department of Clinical Physiology, Nuclear Medicine and PET, Rigshospitalet, Copenhagen University Hospital, Glostrup, Denmark; ^5^Department of Clinical Biochemistry, Rigshospitalet, Copenhagen University Hospital, Glostrup, Denmark; ^6^Odense Patient Data Explorative Network, Odense University Hospital, Institute of Clinical Research, University of Southern Denmark, Odense, Denmark; ^7^Department of Clinical Medicine, Faculty of Health and Medical Sciences, University of Copenhagen, Copenhagen, Denmark; ^8^Clinical Metabolic Physiology, Steno Diabetes Center Copenhagen, Gentofte Hospital, Hellerup, Denmark; ^9^Faculty of Health and Medical Sciences, The Novo Nordisk Foundation Center for Basic Metabolic Research, University of Copenhagen, Copenhagen, Denmark

**Keywords:** exenatide, procollagen type I N-terminal propeptide (PINP), C-terminal cross-linking telopeptide of type I collagen (CTX), bone mineral density, randomized controlled trial

## Abstract

**Background:** Low bone mineral density (BMD) may constitute an underestimated comorbidity in schizophrenia patients undergoing long-term antipsychotic treatment. Glucagon-like peptide 1 (GLP-1) receptor agonists are antidiabetic drugs, which may also affect bone turnover.

**Methods:** In planned secondary analyses of a 3 months, double-blind, randomized, placebo-controlled trial (*n* = 45), we explored effects of the GLP-1 receptor agonist exenatide 2 mg once-weekly (*n* = 23), or placebo (*n* = 22) on bone turnover markers (BTMs) and BMD in chronic, obese, antipsychotic-treated patients with schizophrenia spectrum disorder. Baseline BTMs were compared to sex- and age-adjusted reference values from a Danish population cohort, and *T-* and *Z-*scores were calculated for BMD.

**Results:** In women (*n* = 24), all baseline BTM measurements of procollagen type I N-terminal propeptide (PINP) and C-terminal cross-linking telopeptide of type I collagen (CTX) were within reference values. In men (*n* = 21), 5% displayed lower PINP and 14% displayed lower CTX. One patient displayed BMD *Z-*score < −2, and 23% of patients (17% of women and 29% of men) displayed −2.5 < *T-*scores < –1 indicating osteopenia, but none had osteoporosis. After treatment, PINP decreased at trend level significance (*P* = 0.05), and body mass index BMD increased for L2–L4 (*P* = 0.016). No changes in bone markers were significant after correction for mean prolactin levels.

**Conclusions:** Sex- and age-adjusted measures of bone status in chronic, obese, antipsychotic-treated patients appeared comparable to the reference population. Subtle changes in bone markers during 3 months exenatide treatment may suggest beneficial effects of GLP-1 receptor agonists on bone status in antipsychotic-treated patients, and further studies should consider the potential influence of prolactin.

## Introduction

Antipsychotic medication is the mainstay of treatment of schizophrenia and other psychotic disorders (1). The drug class is generally effective in treating psychotic symptoms, however around 30% of schizophrenia patients do not respond sufficiently (2). Antipsychotics are widely associated with undesirable effects such as extrapyramidal symptoms and dysmetabolism (3, 4), but more recently, osteoporosis and increased risk of bone fractures have also been linked to antipsychotic treatment (3).

Although studies have not consistently reported associations between bone mineral density (BMD) and prolactin levels in antipsychotic-treated patients (5), antipsychotic-induced hyperprolactinaemia has been suggested a causal factor underlying osteoporosis (6). BMD is commonly assessed by dual-energy X-ray absorptiometry (DXA). According to WHO criteria *T-*scores are used as thresholds for osteopenia and osteoporosis. Osteopenia is defined as 1 to 2.5 standard deviations (*SD*) or more below the average value for young healthy subjects of the same sex (−1> *T-*score >−2.5), and osteoporosis is defined as a *T-*score below −2.5 (*T-*score ≤ −2.5) (7). Besides *T-*scores, DXA enables calculation of a *Z-*score, which is a comparison of bone density with a healthy population of the same age and same sex. The reference range for *Z-*scores is ±2. In addition to BMD measurements of bone mass, circulating bone turnover markers (BTMs) can be used to evaluate changes in bone formation and resorption. International consensus guidelines recommend assessment of two BTMs: procollagen type I N-terminal propeptide (PINP) (produced by osteoblasts during bone formation), and C-terminal cross-linking telopeptide of type I collagen (CTX) (released by osteoclasts during bone resorption) (8, 9).

Glucagon-like peptide 1 (GLP-1) receptor agonists are known to induce positive effects on metabolism (10), but the drugs might also affect bone turnover. Animal models have indicated positive effects of the GLP-1 receptor agonists exendin-4 and liraglutide on bone metabolism (11–13). These findings have motivated translational efforts aiming to investigate the potential benefits of GLP-1 receptor agonists on bone status in humans. Treatment with liraglutide has been shown to increase bone formation in body weight-reduced obese women when compared to placebo (14). Conversely, studies of type 2 diabetes patients have indicated that GLP-1 receptor agonists have no effect on bone metabolism or fracture risk (15, 16).

The current study comprises planned secondary analyses of the “TAO study”: Treatment of antipsychotic-associated obesity with a GLP-1 receptor agonist (17–20). The TAO study was an investigator-initiated, double-blind, randomized, placebo-controlled trial, investigating the effects of 3 months treatment with the GLP-1 receptor agonist exenatide 2 mg once-weekly in chronic obese, antipsychotic-treated patients with schizophrenia spectrum disorder. First we compared baseline BTMs with the Danish Health 2006 study cohort as reference population (21), and we calculated BMD *T-* and *Z-*scores. Next, we compared baseline PINP, CTX and BMD with end-of-trial measures aiming to unravel potential beneficial effects of exenatide on BTMs and BMDs.

## Methods

Details of the “TAO study” have previously been reported (17–20). Below, key methodology, experimental procedures and analyses are outlined.

### Study Population and Procedures

Inclusion criteria included clinically stable schizophrenia spectrum patients (ICD-10 diagnoses F20.x and F25.x); treatment with minimum one antipsychotic drug; age 18 to 65 years; obesity (BMI ≥ 30 kg/m^2^). Exclusion criteria included substance dependence, diabetes (any type), severe somatic disease, pregnancy and breastfeeding. Patients were randomized to either receive injections of 2 mg exenatide once-weekly (Bydureon®, AstraZeneca AB, Södertälje, Sweden) or placebo. We used the solvent from the Bydureon® kit as placebo. Unblinded trial staff otherwise not involved in the study performed the subcutaneous injections of exenatide or placebo ensuring 100% medication adherence. Both groups were assessed with biochemical analyses and DXA measurements at trial initiation and after 3 months (12–16 weeks) (17).

### Biochemical Analyses

All biochemical analyses were performed at the Department of Clinical Biochemistry, Rigshospitalet, Copenhagen, Denmark. Since CTX (8) and prolactin (22) are influenced by diurnal variations, all blood samples were collected in the morning before food intake (fasting >8 h).

### Bone Mineral Density

Patients underwent DXA examinations on a Lunar Prodigy whole-body scanner (GE Medical Systems, Madison, Wisconsin, USA). As input for statistical analyses, we calculated averages of the left and right femoral neck measurements and the left and right total femur measurements. *T-*score and *Z-*score were not calculated for patients below 20 years of age. Likewise, we calculated standardized BMD values from the raw data as described by Fan et al. (23).

### Statistical Analyses

To enable comparison with the sex and age intervals obtained from the background population cohort (21), we split patients into men and women and compared baseline levels of PINP, CTX, and BMD for each group and age interval, separately. Baseline demographic and clinical variables were tested with independent *t*-tests for continuous data, and χ^2^-test for nominal data. Non-normally distributed BTM and BMD values were transformed by logarithm or square root to achieve normal distribution. All outcomes were initially analyzed without covariates by two-way repeated measures ANOVA. Next, analyses were repeated with mean prolactin level [(baseline + follow-up)/2] as a covariate to evaluate the potential effect of prolactin. We *a priori* decided to repeat analyses after excluding patients with baseline values, which could indicate pre-study disturbance of bone metabolism (vitamin D < 30 nmol/L and/or parathyroid hormone (PTH) >7.63 pmol/L) (24). IBM SPSS Statistics Version 22 (IBM Corp. for Windows, Armonk, NY) was used for statistical analyses. The significance level was set to 0.05, and all tests were two-tailed.

## Results

### Demographic and Clinical Data

In total, 45 patients were included in the baseline analyses. Twenty patients in the exenatide-treated group and 20 patients in the placebo-treated group completed the trial (Supplementary Figure 1). At baseline we found no significant group differences in age, sex, ethnicity, illness duration, education, body weight, BMI, diagnosis or antipsychotic medication. However, we found a higher proportion (*p* = 0.02) of current smokers in the exenatide group (Supplementary Table 1). After 3 months of exenatide or placebo treatment patients lost 2.3 kg with no significant difference between groups (18, 20).

### Comparison of Baseline Bone Turnover Markers to Reference Values

**Figure 1:**
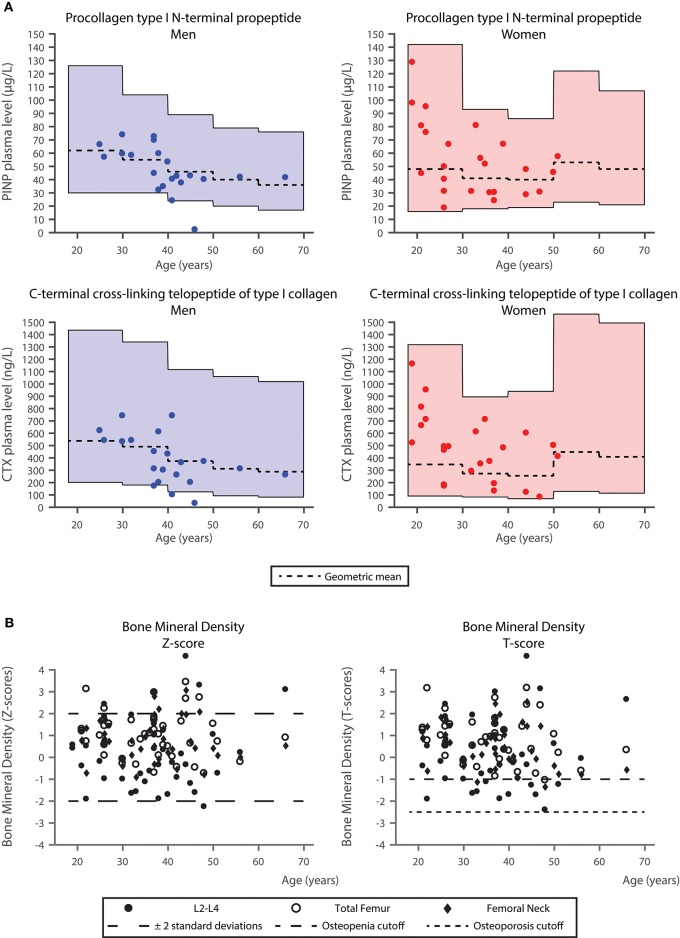
Comparison of bone turnover markers and bone mineral density in the study cohort with the background population. **(A)** Procollagen type I N-terminal propeptide (PINP) and C-terminal cross-linking telopeptide of type I collagen (CTX) levels in the study cohort with reference ranges obtained from the background population (21). Geometric means for the reference intervals are plotted. **(B)** Bone mineral density values of the lumbar spine L2–L4, left and right femoral neck, as well as left and right total femur (acquired using CORE software [version 14.1]). Age is plotted against *Z-*scores (average values for a healthy population of the same age and same sex) (left panel) and *T-*scores (average values for young healthy subjects of the same sex) (right panel). In the plot of *Z-*scores, ±2 standard deviations are presented. In the plot of *T-*scores, the cutoffs for osteopenia and osteoporosis are presented (*T-score* = −1 *SD* and *T-score* = −2.5 *SD*).

PINP and CTX concentrations were within the age-adjusted reference range (21) for all women (24 of 24). One of 21 male patients (5%) had lower PINP, and three male patients (14%) had lower CTX levels than the corresponding age-adjusted reference range (Figure 1A).

### Comparison of Baseline Bone Mineral Density to Reference Values

One hundred and fourteen (86%) out of the total 132 DXA measurements corresponded to *Z-*scores between ±2. One patient had a *Z-*score (L2–L4) < −2. In both the L2–L4 and the total femur measurements five patients (11%) had *Z-*scores above 2, whereas in the femoral neck BMD measurement four patients (9%) had *Z-*scores above 2 (Figure 1B). Ten patients (23%), [4 women (17%), and 6 men (29%)] had *T-*scores < −1 indicating osteopenia. No patients had osteoporosis (*T-*scores ≤ −2.5) (Figure 1B).

### Effect of Exenatide on Biomarkers of Bone Turnover and Bone Mineral Density

After 3 months we observed numerical reductions in levels of both PINP and CTX in the exenatide-treated group, whereas the levels in the placebo-treated group numerically increased. For PINP, we found a time × group interaction (i.e., a treatment effect) at trend-level significance (*p* = 0.05), however, when prolactin was included as a covariate this trend-level observation was no longer present. For CTX, we observed no significant interactions. Apart from a trend-level increase in osteocalcin over time in both groups, analyses on other bone-related biomarkers were non-significant (Table 1).

**Table 1:** Effect of exenatide and placebo on biomarkers related to bone metabolism and on bone mineral density.

**Blood marker**	**Exenatide (*n* = 20) Mean ± *SD* [Range]**	**Placebo (*n* = 20) Mean ± *SD* [Range]**	**Time *p*-value**	**Group *p*-value**	**Time x Group *p*-value (No covariance)**	**Time x Group *p*-value (Prolactin covariance)**
C-terminal cross-linking telopeptide of type I collagen (ng/L) $						
Baseline	409.0 ± 229.6 [170–1160]	475.5 ± 265.7 [30-950]	0.93	0.19	0.15	0.25
End of trial	357.0 ± 171.9 [80–780]	514.5 ± 292.5 [60–1420]				
Procollagen type I N-terminal propeptide (μg/L)						
Baseline	49.3 ± 23.0 [18.5–128.4]	52.4 ± 24.9 [2.0-97.7]	0.52	0.27	0.05	0.39
End of trial	46.1 ± 20.5 [17.2–104.3]	58.7 ± 24.4 [29.2–111.9]				
Bone-specific alkaline phosphatase (μg/L)						
Baseline	20.9 ± 8.3 [11.3–47.1]	22.2 ± 10.3 [8.2–50.8]	0.65	0.55	0.61	0.26
End of trial	20.8 ± 7.3 [11.3–37.5]	22.8 ± 9.7 [8.7–48.6]				
Osteocalcin (μg/L) § Baseline End of trial	14.3 ± 7.6 [2.0–37.7] 15.4 ± 5.9 [8.0–33.5]	16.1 ± 8.0 [6.2–39.0] 17.8 ± 8.5 [8.7–42.4]	0.05	0.28	0.71	0.40
Osteocalcin/CTX ratio § Baseline End of trial	40.3 ± 21.1 [4.3–96.5] 54.1 ± 39.2 [18.7–176.7]	51.5 ± 52.4 [19.2–256.7] 47.0 ± 40.7 [14.6–188.3]	0.28	0.86	0.11	0.24
Parathyroid hormone (pmol/L) $ Baseline End of trial	4.30 ± 3.30 [1.0–14.7] 3.71 ± 1.88 [1.1–9.6]#	4.15 ± 1.90 [1.1–10.8]# 4.08 ± 1.81 [1.5–9.3]#	0.32	0.75	0.30	0.31
Prolactin (mIU/L) § Baseline End of trial	369.6 ± 397.3 [24.1–1484.0] 357.7 ± 404.0 [20.9–1416.0]	435.3 ± 434.9 [34.2–1614.0] 372.8 ± 393.7 [30.4–1502.0]	0.11	0.74	0.42	–
Vitamin D (nmol/L) Baseline End of trial	62.9 ± 33.8 [8.0–126.0] 69.0 ± 37.7 [16.2–135.0]	70.4 ± 33.6 [14.2–130.0] 72.8 ± 31.3 [17.0–136.0]	0.14	0.59	0.51	0.67
**Dual-energy X-ray absorptiometry—Bone mineral density (g/cm**^**2**^**)**	**Exenatide (*****n*** **=** **20) Mean** **±** ***SD*** **[Range]**	**Placebo (*****n*** **=** **19) Mean** **±** ***SD*** **[Range]**	**Time** ***p*****-value**	**Group** ***p*****-value**	**Time x Group** ***p-*****value (No covariance)**	**Time x Group** ***p*****-value (Prolactin covariance)**
L2–L4 Baseline End of trial	1.23 ± 0.19 [0.90–1.68] 1.24 ± 0.19 [0.93–1.67]	1.13 ± 0.16 [0.92–1.49] 1.12 ± 0.16 [0.88–1.48]	0.576	0.055	0.016^*^	0.057
Femoral neck Baseline End of trial	1.05 ± 0.13 [0.82–1.28] 1.04 ± 0.13 [0.76–1.26]	1.00 ± 0.10 [0.80–1.21] 1.00 ± 0.10 [0.82–1.16]	0.125	0.223	0.576	0.720
Total femur Baseline End of trial	1.12 ± 0.12 [0.90–1.38] 1.13 ± 0.11 [0.89–1.40]	1.08 ± 0.11 [0.90–1.35] 1.08 ± 0.11 [0.90–1.35]	0.976	0.212	0.419	0.070

Bone mineral density was measured with dual-energy X-ray absorptiometry scanning. All outcomes were initially analyzed without covariates by two-way repeated measures ANOVA, where the between-subject factor, i.e. exenatide vs placebo, was denoted “Group,” and the within-subject factor between time points was denoted “Time.” A significant “Time × Group interaction” would indicate a difference in response between the two treatment groups. Results from ANOVA/ANCOVA are corrected for age and sex. P-values are rounded to two decimals and significant p-values are shown with an asterisk (^*^). The table is based on data from patients, who completed the trial.

*# One observation missing. $ Square root-transformed to obtain normal distribution. § Natural logarithm-transformed to obtain normal distribution. Alkaline phosphatase and parathyroid hormone (PTH) were measured on the Vitros 5.1FS or the Vitros 5600 chemistry analyzer (Ortho Clinical Diagnostics, Raritan, NJ, USA) and total 25-hydroxycholecalciferol (vitamin D) was measured on the Cobas e411 analyzer (Roche Diagnostics, Rotkreuz, Switzerland). All three assays are electro-chemiluminescence binding assays. Prolactin was measured using an immunofluorimetric assay on the BRAHMS Kryptor Compact Plus analyzer (Thermo Scientific, Hennigsdorf, Germany). Plasma PINP, plasma CTX, plasma osteocalcin, and serum bone-specific alkaline phosphatase were measured with chemiluminescence immunoassays using the automated analyzer, iSYS (Immunodiagnostic Systems plc, Tyne and Wear, UK) according to the manufacturer's instructions*.

Analyses of BMD data (Table 1) revealed a significant time × group interaction (*p* = 0.016) in the L2–L4, indicating higher BMD after exenatide treatment and lower BMD after placebo. After correction for the mean prolactin level, this interaction was only significant at trend-level (*p* = 0.057). The remaining analyses of BMD were not significant.

Exclusion of four patients with suspected pre-study disturbance of bone metabolism did not change the significance levels of the above results.

## Discussion

The current analyses of bone status in chronic, obese, non-diabetic, antipsychotic-treated schizophrenia spectrum patients indicated that most patients had BTMs, i.e., PINP and CTX levels within the reference ranges obtained from a Danish background population (21). On the contrary, our BMD measurements indicated that 23% of patients had osteopenia. However, in men younger than 50 years of age and premenopausal women, *T-*scores are typically not used for BMD (25), rather *Z-*scores are preferred. Nevertheless, a meta-analysis of prevalence of low bone mass in schizophrenia patients reported an even higher prevalence of osteopenia which was present in both patients and controls (around 40%) (26). This could be explained by the fact that the patients included in our trial were markedly obese (mean BMI 38.8 kg/m^2^), and obesity is generally associated with increased BMD. Paradoxically, obesity has also been associated with an increase in fracture risk (27). Hence, the seemingly unaffected BMD observed in our patient sample may still render the patients at an increased risk of fractures, but the association between BMI and fracture risk is complex (28), and fracture risk was beyond what could be assessed from the current data.

The mean age of our patients was 35.8 years, and with one exception, all sex and age-adjusted BMD *Z-*score measurements were within the normal range. In fact, five patients (12%) had *Z-*scores above 2 in L2–L4 and total femur measurements, and four patients (9%) had *Z-*scores above 2 in the femoral neck BMD measurement (Figure 1B). Therefore, in contrast to our expectations, the current comparative baseline analyses do not lend overall support to the emerging concern of markedly compromised bone status in chronic schizophrenia patients. As noted above the presence of marked obesity may partly explain these findings.

We observed that treatment with exenatide resulted in a trend-level reduction in PINP, and a significant increase in the BMD measurement of L2–L4. The reduction in PINP in the exenatide-treated group contrasts a previously reported increase of PINP in obese women, who experienced a 12% body weight reduction after 12 months of liraglutide treatment (14). In our 3 months study, patients experienced a weight loss of 2.3 kg corresponding to a subtle reduction in body weight of around 2%. Based on these placebo-controlled studies it could appear that GLP-1 receptor agonists may affect PINP, however, the directionality of this change may be influenced by antipsychotic exposure or by concurrent changes in body weight. Additionally, the two GLP-1 receptor agonists liraglutide and exenatide may also affect levels of PINP differently.

Finally, in our study the potential effect of exenatide on bone markers did not remain significant after correction for mean prolactin levels. The limited sample size and large variability in prolactin level render the impact of this finding unclear. Although a previous study did not find correlation between prolactin levels and BMD measures (5), modulation of the dopamine system by GLP-1 receptor agonism has previously been suggested (29). Nevertheless, our current observation of a potential interplay between prolactin levels and effect of GLP-1 receptor agonists, suggests that correction for prolactin in future studies of antipsychotic-treated patients should be considered.

The current study has some limitations. The 3 months study period, and the relatively young (with respect to bone status), and non-diabetic sample compromise the inferences which can be drawn from the present data. Firstly, our patients and the population cohort were not matched on BMI (30). Moreover, we intentionally included a naturalistic trial population (18), which is reflected in the broad medication profiles (Supplementary Table 1). To this end, individual antipsychotic compounds may affect prolactin levels (22, 31, 32), and bone status differentially, but the current data did not allow for separating effects of specific antipsychotics. Finally, patients were not instructed to keep their level of physical activity stable and refrain from taking vitamin D supplements during the trial, and we were therefore unable to control for these potential confounders.

In conclusion, these planned secondary analyses of the TAO study showed that sex and age-adjusted measures of bone status were comparable to the Danish reference population. Subtle changes in bone markers over a 3 months treatment course with the GLP-1 receptor agonist exenatide may suggest beneficial effects of GLP-1 on bone status in antipsychotic-treated, obese patients, which may relate to GLP-1-induced changes in prolactin levels.

## Ethics Statement

This study was approved by the National Committee on Health Research Ethics (project no. 36378), the Danish Health and Medicines Authority (EudraCT no. 2012-005404-17) and The Danish Data Protection Agency (project no. RHP-2012-027). The study was registered at clinicalTrials.gov (NCT01794429). The Good Clinical Practice (GCP) Unit at Copenhagen University Hospital monitored the trial according to ICH-GCP guidelines.

All referred patients received both an oral and a written description of the TAO trial, and all were screened for eligibility by the principal investigator (PLI). All patients approved participation by written informed consent prior to enrolment.

## Author Contributions

All authors fulfill authorship criteria of the ICMJE by substantial contribution to the conception and design, to acquisition of data, or to the analysis and interpretation of the data. FK and BE contributed conception and design of the study. PI acquired the data. BB, PI, and NB organized the database. RE, BB, NB, and BE performed the statistical analysis. RE and BE wrote the first draft of the manuscript. RE, PI, UA, NJ, and FK wrote Methods section of the manuscript. RE, BB, NB, NJ, and BE wrote Results section of the manuscript. All authors contributed to manuscript revision, read, and approved the submitted version. The trial was investigator-initiated and data analysis was conducted without influence from the pharmaceutical industry. We also affirm that there was no editorial direction or censorship from any pharmaceutical company.

### Conflict of Interest Statement

BB became a full-time employee at Novo Nordisk after completion of the clinical study. NB became a full-time employee at H. Lundbeck A/S after completion of the clinical study. FK has received lecture fees from, is part of Advisory Boards of and/or has consulted for AstraZeneca, Boehringer Ingelheim, Carmot Therapeutics, Eli Lilly, Merck Sharp & Dohme, Novo Nordisk, Norgine, Sanofi, and Zealand Pharma. BE has received lecture fees from and/or is part of Advisory Boards of Bristol-Myers Squibb, Eli Lilly and Company, Janssen-Cilag, Otsuka Pharma Scandinavia, and Takeda Pharmaceutical Company. The remaining authors declare that the research was conducted in the absence of any commercial or financial relationships that could be construed as a potential conflict of interest.
