# Supplementary material for: Bone Status in Obese, Non-diabetic, Antipsychotic-Treated Patients, and Effects of the Glucagon-Like Peptide-1 Receptor Agonist Exenatide on Bone Turnover Markers and Bone Mineral Density
Source: Front Psychiatry. 2019 Jan 28;9:781. doi: 10.3389/fpsyt.2018.00781 (PMC6360839; doi:10.3389/fpsyt.2018.00781)
Supplement: Supplementary file 1 [file Table_1.pdf]

Supplementary Table 1. Demographic and clinical characteristics of the patients at baseline.

	Exenatide (n=23)	Placebo (n=22)	p-value
**Age (year) – mean ± SD [range]**	37.1 ± 10.6 [19-65]	34.5 ± 10.1 [19-56]	0.40
**Sex (male / female) – no. (%)**	11 (48) / 12 (52)	10 (45) / 12 (55)	0.87
**Ethnicity (caucasian / mongolian) – no. (%)**	21 (91) / 2 (9)	19 (86) / 3 (14)	0.60
**Illness duration (weeks) – mean ± SD [range]**	689 ± 463 [38-1404]#	578 ± 426 [26-1404]	0.41
**Education (years) – mean ± SD [range]**	12.6 ± 2.9 [8-18]	12.1 ± 2.6 [7-20]	0.51
**Current smoker – no. (%)**	7 (30)	1 (5)	0.02
**Body weight (kg) – mean ± SD [range]**	117.1 ± 16.7 [84.4-150.7]	110.6 ± 17.7 [88.2-149.7]	0.21
**BMI (kg/m^2) – mean ± SD [range]**	39.2 ± 3.8 [31.0-48.0]	38.4 ± 6.1 [30.1-55.1]	0.59
**Diagnosis**			
**Schizophrenia, F20.X – no. (%)**	21 (91)	20 (91)	-
**Schizoaffective disorders, F25.X – no. (%)**	2 (9)	2 (9)	
**Antipsychotic medication**			-
**Typical monotherapy – no. (%)**	1 (4)	1 (5)	0.97
**Atypical monotherapy – no. (%)**	15 (65)	11 (50)	0.30
**Polypharmacy – no. (%)**	7 (30)	10 (45)	0.30

One observation missing.
