# Supplementary figures and images for: Bone Status in Obese, Non-diabetic, Antipsychotic-Treated Patients, and Effects of the Glucagon-Like Peptide-1 Receptor Agonist Exenatide on Bone Turnover Markers and Bone Mineral Density
Source: Front Psychiatry. 2019 Jan 28;9:781. doi: 10.3389/fpsyt.2018.00781 (PMC6360839; doi:10.3389/fpsyt.2018.00781)

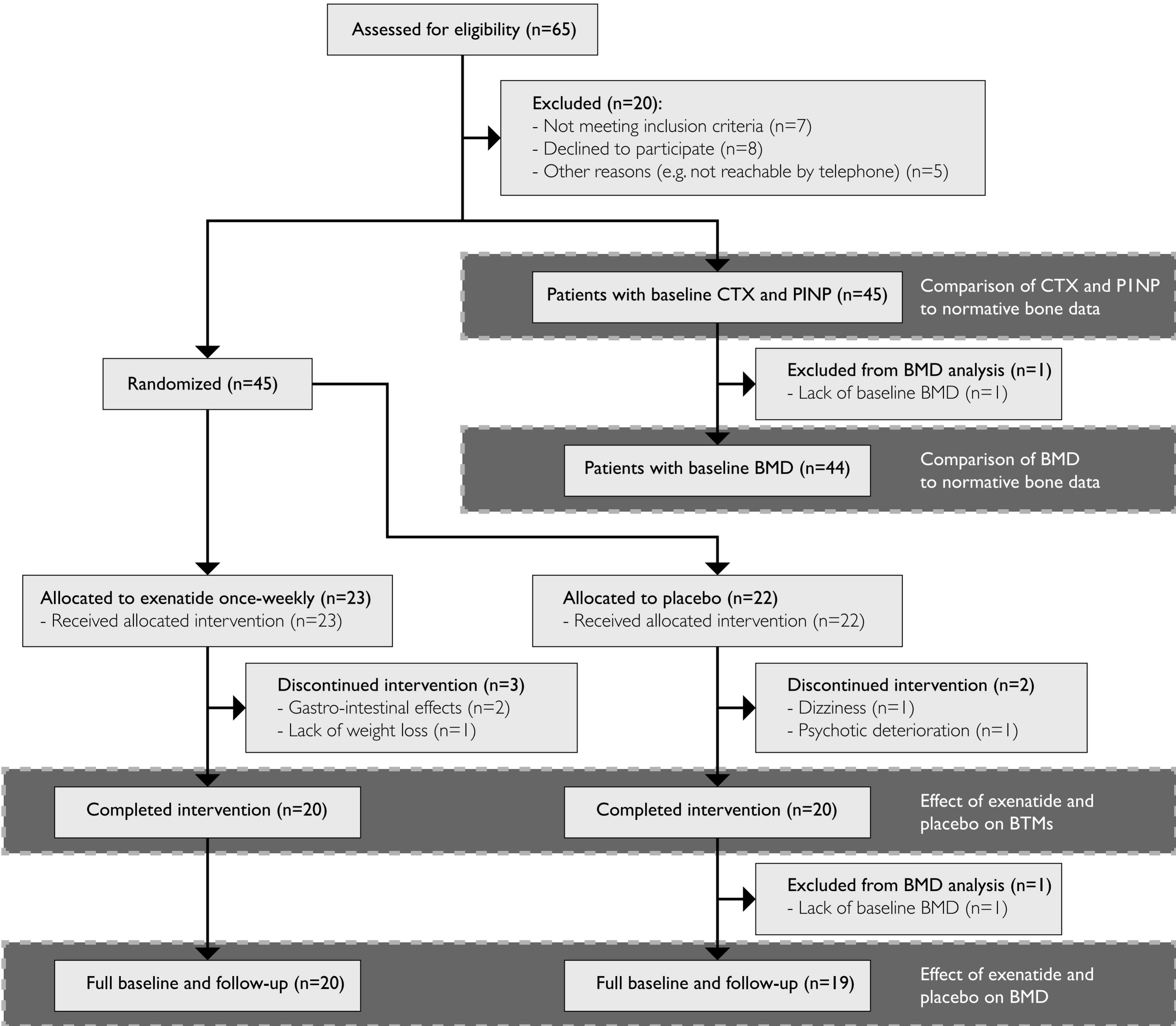

Supplementary Figure I. CONSORT flowchart of the trial.

Supplement: Supplementary file 2 [file Image_1.pdf]
